# Transcriptome and Oxylipin Profiling Joint Analysis Reveals Opposite Roles of 9-Oxylipins and Jasmonic Acid in Maize Resistance to Gibberella Stalk Rot

**DOI:** 10.3389/fpls.2021.699146

**Published:** 2021-09-07

**Authors:** Qing Wang, Yali Sun, Fang Wang, Pei-Cheng Huang, Yinying Wang, Xinsen Ruan, Liang Ma, Xin Li, Michael V. Kolomiets, Xiquan Gao

**Affiliations:** ^1^State Key Laboratory for Crop Genetics and Germplasm Enhancement, Nanjing Agricultural University, Nanjing, China; ^2^Jiangsu Collaborative Innovation Center for Modern Crop Production, Nanjing Agricultural University, Nanjing, China; ^3^College of Agriculture, Nanjing Agricultural University, Nanjing, China; ^4^Department of Plant Pathology and Microbiology, Texas A&M University, College Station, TX, United States

**Keywords:** 9-oxylipins, lipoxygenase, ketols, transcriptome, *Fusarium graminearum*

## Abstract

Gibberella stalk rot caused by *Fusarium graminearum* is one of the devastating diseases of maize that causes significant yield losses worldwide. The molecular mechanisms regulating defense against this pathogen remain poorly understood. According to recent studies, a major oxylipin hormone produced by 13-lipoxygenases (LOX) namely jasmonic acid (JA) has been associated with maize susceptibility to GSR. However, the specific roles of numerous 9-LOX-derived oxylipins in defense against Gibberella stalk rot (GSR) remain unexplained. In this study, we have shown that disruption of a 9-LOX gene, *ZmLOX5*, resulted in increased susceptibility to GSR, indicating its role in defense. To understand how *ZmLOX5* regulates GSR resistance, we conducted transcriptome and oxylipin profiling using a *zmlox5-3* mutant and near-isogenic wild type B73, upon infection with *F. graminearum*. The results showed that JA biosynthetic pathway genes were highly up-regulated, whereas multiple 9-LOX pathway genes were down-regulated in the infected *zmlox5-3* mutant. Furthermore, oxylipin profiling of the mutant revealed significantly higher contents of several jasmonates but relatively lower levels of 9-oxylipins in *zmlox5-3* upon infection. In contrast, B73 and W438, a more resistant inbred line, displayed relatively lower levels of JAs, but a considerable increase of 9-oxylipins. These results suggest antagonistic interaction between 9-oxylipins and JAs, wherein 9-oxylipins contribute to resistance while JAs facilitate susceptibility to *F. graminearum*.

## Introduction

Gibberella stalk rot caused by *Fusarium graminearum* (*teleomorph Gibberella zeae*) severely impacts maize (*Zea mays*) production worldwide, leading to dramatic yield losses (Mueller and Wise, 2015). Major efforts are devoted to breeding Gibberella stalk rot (GSR)-resistant varieties because the application of certain agronomic practices and fungicides in GSR control are either less effective or harmful to the environment.

A few reports are dealing with GSR resistance mechanisms. For instance, disruption of *ZmCCT*, a candidate gene for *qRfg1*, reduced *Fusarium* spp. resistance (Wang et al., 2017), while *ZmAuxRp1*, a candidate gene for *qRfg2*, exerts its function *via* balancing the growth and defense by regulating DIMBOA content upon *F. graminearum* infection (Ye et al., 2019). Despite these findings, the complex mechanisms underlying maize resistance to GSR remain largely unexplored.

Increasing reports have suggested that plant oxylipins play crucial roles in defense response to pathogens, serving as signaling molecules or having direct toxicity for microbes (Howe and Schilmiller, 2002; Famer et al., 2003; Deboever et al., 2020). Oxylipins represent a large family of oxidized fatty acids, mainly generated from linoleic acid (C18:2) or α-linolenic acid (C18:3), the catalysis of which is initiated by lipoxygenases (LOXs) and α-dioxygenases (DOX) (Blee, 2002; Feussner and Wasternack, 2002; Andreou et al., 2009). According to the oxidized position of carbon (C), LOXs are divided into 9- and 13-LOXs (Feussner and Wasternack, 2002; Schneider et al., 2007). Jasmonic acid (JA) biosynthesis is initiated by oxidation of α-linolenic acid by 13-LOXs followed by the action of allene oxide synthase (AOS) and allene oxide cyclase (AOC) resulting in the production of a JA precursor, 12-oxo-phytodienoic acid (12-OPDA). Afterward, 12-OPDA is reduced by 12-OPDA reductase (OPR) followed by three rounds of beta-oxidation to produce (+)-7-iso-JA (Wasternack, 2007; Borrego and Kolomiets, 2016). Then, (+)-7-iso-JA is conjugated to isoleucine by the JAR1 enzyme resulting in the synthesis of the biologically active JA-Ile. On the other hand, 9-LOXs catalyze the formation of a series of 9-oxylipins, such as (9*S*, 10*E*, 12*Z*)-9-hydroperoxy-10, 12-octadecadienoic acid (9*S*-HPODE) and others (Feussner and Wasternack, 2002; Howe and Schilmiller, 2002).

It has been well-documented that 13-oxylipins, including JAs and their derivatives, play diverse roles in plant-pathogen interactions (Bate and Rothstein, 1998; Birkett et al., 2000; Turner et al., 2002; Wasternack and Feussner, 2018; Deboever et al., 2020). For instance, two maize 13-LOXs, e.g., ZmLOX10 and ZmLOX11, were shown to be highly induced by pathogen infection (Nemchenko et al., 2006). Disruption of *ZmOPR7* and *ZmOPR8*, two key genes responsible for JA biosynthesis in maize, resulted in complete loss of immunity against soil-borne necrotrophic pathogens, e.g., *Pythium* spp. (Yan et al., 2012), and *Fusarium verticillioides* (Christensen et al., 2014), but increased resistance to the hemibiotrophic *Colletotrichum graminicola* (Gorman et al., 2020). Furthermore, jasmonic acid-isoleucine (JA-Ile) was induced in response to various biotic stresses (Howe et al., 2018). Interestingly, accumulating evidence suggested that the JA precursor, 12-OPDA, could function as a signaling molecule independent of JA-Ile. Most recently, 12-OPDA and α-ketol of octadecadienoic acid (KODA) were identified as critical xylem-mobile signaling molecules to activate induced systemic resistance (ISR) triggered by maize root colonization of the beneficial fungus, *Trichoderma virens* (Wang et al., 2020).

The functions of the majority of 9-oxylipins in plant biotic stress responses are largely unexplored compared with JAs (Deboever et al., 2020). Nevertheless, increasing evidence showed that 9-oxylipins also play a role in plant-pathogen interactions. For example, disruption of a tobacco 9-LOX gene led to the loss of hypersensitive reaction (HR)-mediated resistance to *Phytophthora parasitica* (Rance et al., 1998). This result is likely due to the reduced levels of 9-LOX derived colnelenic and colnelenic acids (Fammartino et al., 2007). Furthermore, 9-KOT was strongly induced upon infection by *Pseudomonas syringae* pv. tomato (*Pst*) in Arabidopsis (Vicente et al., 2012). In maize, ZmLOX1, 2, 3, 4, 5, and 12 comprise the 9-LOX category (Christensen et al., 2014; Borrego and Kolomiets, 2016). Functional analysis of *zmlox3-4* mutant showed that the oxylipins produced by this specific 9-LOX are required for fungal pathogenesis by *F. verticillioides* and other pathogens of leaf, stem, and root (Gao et al., 2007; Isakeit et al., 2007; Pathi et al., 2020), Increased resistance of *zmlox3-4* mutant to *F. verticillioides* was explained by the over-expression of other 9-LOXs, *ZmLOX4, ZmLOX5*, and *ZmLOX12*, as *zmlox4, zmlox5*, and *zmlox12* mutants were more susceptible to *F. verticillioides*, due to decreased JA levels in response to infection (Christensen et al., 2014; Battilani et al., 2018). However, the roles of 9-oxylipins in maize resistance to GSR remain unexplored.

In this study, we tested *zmlox5-3* mutant and its corresponding wild type (WT) maize line B73, for resistance to GSR. Disruption of *ZmLOX5* resulted in increased GSR disease severity. RNA-seq transcriptome analyses revealed that the mutant responded to *F. graminearum* infection by increased transcript accumulation of the genes related to JA synthesis and signal transduction pathways yet decreased expression of the 9-LOX pathway genes in *zmlox5* mutant compared with WT. In agreement with this, *zmlox5-3* accumulated significantly higher levels of JA-Ile and other JAs, but lower levels of several 9-oxylipins upon infection. Thus, it is concluded that ZmLOX5-produced 9-oxylipins contribute to GSR resistance, whereas JAs appear to facilitate *F. graminearum* virulence.

## Materials and Methods

### Plant Material and Fungal Strain

The seeds of maize lines W438, B73, and the transposon element-insertional mutant zmlox5-3 of B73, provided by Prof. Kolomiets laboratory, were sown in the soil in PVC cylinders (5 cm in diameter and 20 cm in depth) and grown under the condition of day/night of 14/10 h photoperiod at 26 ± 2°C and 75% humidity on a growth shelf. Two-week-old seedlings were transferred to a culture room for phenotypic assay.

*Fusarium graminearum* strain 0609, provided by Prof. Mingliang Xu, China Agricultural University, was initially cultured on potato dextrose agar (PDA) media for 7 days and then maintained at 4°C until use. There were 10 pieces of 0.5 cm^3^ PDA plug freshly excised from the agar plate, then cultured in 40 ml fresh mung bean broth on a rotary shaker at 25°C and 200 rpm for 3–4 days. The concentration of spore suspension was adjusted to 1 × 10^6^ macroconidia per ml supplemented with Tween-20 to a final concentration at 0.001%.

### Inoculation Assay, Sampling, and Disease Phenotyping

The fungal inoculum and artificial inoculation of seedlings were conducted according to our previous study (Sun et al., 2018). Prior to inoculation, maize seedlings were lawn down in a plastic square tray (100 × 80 × 10 cm) and a hole was punched by a 1 ml sterile syringe needle on the middle of the stem of the seedling. Afterward, 20-μl spore suspension was carefully dropped onto the wounded site and the square tray was sealed by saran wrap to keep the humidity. The culture condition was maintained at 24 ± 2°C, with 14 h light/10 h dark period and 200 lux light intensity.

For RNA-seq and qRT-PCR analysis, 12 uninfected or infected stems from each line were collected at 0, 12, 24, and 48 h after infection (hai) respectively, then stored in –80°C for further study. For fungal biomass quantification, 10 infected maize stems from each line were collected at 72 hai. Gibberella stalk rot disease symptoms were scored using 20 seedlings per genotype at 3 days post-inoculation (dpi) according to a five-level seedling GSR scale, ranging from 1 (most resistant) to 5 (most susceptible) (Sun et al., 2018). The statistical analysis was performed by using SPSS (14.0) (IBM, Stanford, USA) for ANOVA and Student's *t*-test, with the significance level of *p* ≤ 0.05.

### Isolation of Genomic DNA and Quantification of Fungal Biomass

The isolation of genomic DNA (gDNA) was carried out using maize stems by a CTAB (cetyl trimethylammonium bromide)-based method described by the study of Brandfass and Karlovsky (2008). The gDNA of the inoculated seedling stem was diluted to a 50 ng/μl working solution for the quantification of stem and fungal biomass. The gDNA of the non-inoculated stem was used as an external DNA standard in the reference gene-based qRT-PCR analysis, by diluting to a series of concentrations, i.e., 100, 50, 25, 6.25, 1.5625, and 0.39 ng of DNA per microliter.

For fungal biomass quantification, Potato dextrose broth was inoculated with 5 ml of 1 × 10^6^ macroconidia suspension of *F. graminearum* 0609 and incubated on a rotary shaker at 25°C for 3–5 days. The mycelium was harvested and freeze-dried, and gDNA was isolated as described by the study of Brandfass and Karlovsky (2008), as *Fusarium* standard in the reference gene-based qRT-PCR. The genomic DNA was diluted to a series of concentrations at 50, 10, 5, 1, 0.5, and 0.1 ng of *F. graminearum* DNA per μl and kept at −20°C until use.

The qRT-PCR was performed on a CFX96 real-time PCR system (Bio-rad, Hercules, CA, USA). Primers of *F. graminearum* Tubulin and maize β*-Tubulin 4* gene (Zm00001d013612) used to quantify *F. graminearum* and maize biomass, respectively (listed in Supplementary Table 1). The amplification mix consisted of 1 μl of template DNA, 5 μl of universal SYBR green master (Monad, Suzhou, China), 2 μl of double-distilled water, and 1 μl each of forward and reverse primer (10 pmol/μl). The qRT-PCR was performed according to the description of the study by Wang et al. (2015), with a cycle at initial denaturation step at 94°C for 1.5 min, followed by 35 cycles of 94°C for 30 s, 60°C for 45 s, and 72°C for 45 s, and elongation at 72°C for 5 min. The average amount of *F. graminearum* in maize stem and the infection percentage of stem samples (relative fungal biomass) were calculated based on the description in the study conducted by Brunner et al. (2009). The infection index was calculated as the average of three biological replicates.

### RNA-Seq and qRT-PCR Analysis

Total RNA was extracted from *F. graminearum*-inoculated and uninoculated B73 and *zmlox5-3* seedling stems at different time points using Trizol reagent (Invitrogen, CA, USA), then purified with the RNeasy Mini Kit (Qiagen, Hilden, Germany). Following the quality control of RNA samples using Illumina TruSeq RNA library prep kit v2, RNA sequencing was performed with Illumina HiSeq 2000 (Illumina Inc., Berry, Beijing).

Paired-end RNA sequence reads of 150 bases were generated, and all clean reads were mapped to the reference genome of maize inbred B73 (RefGen_V4) and the reference genome dataset of *F. graminearum* (FGS 5b) using HISAT2 (version 2.2.1.0) (https://daehwankimlab.github.io/hisat2/). The gene expression values were normalized as gene counts. To identify differentially expressed genes (DEGs), DESeq2 (version 1.32.0) (https://bioconductor.org/packages/release/bioc/html/DESeq2.html) was used with the threshold of a false-discovery rate (FDR) <0.05. DEGs were annotated according to maize genome from NCBI and were subjected to Gene Ontology (GO) enrichment (www.geneontology.org) (Ashburner et al., 2000) and KEGG pathway analyses (www.genome.jp/kegg) (Kanehisa et al., 2016). Three independent biological replicates were conducted with each sample containing at least six seedlings.

To verify the dynamic change of key genes in the 9- and 13-LOX pathway of three genotypes, 20 genes were selected to conduct quantitative RT–PCR analysis using the CFX96 real-time PCR system (Bio-rad, Hercules, CA, U.S.A.) (Supplementary Table 1). Complementary DNA (cDNA) synthesis was carried out using 1 μg of total RNA. The PCR reaction mix consisted of 1 μl of template cDNA, 5 μl of universal SYBR green master, 2 μl of double-distilled water, and 1 μl each of forward and reverse primer (10 pmol/μl). PCR was performed according to the following protocol: initial denaturation for 10 min at 94°C, followed by 44 cycles with 45 s at 94°C, 45 s at 60°C, and 45 s at 72°C, and the final elongation at 72°C for 5 min. Fluorescence was determined during the annealing step of each cycle. Following amplification, the melting curves were acquired by heating the samples to 95°C for 15 s, cooling to 60°C for 1 min, and then slowly increasing the temperature from 60 to 95°C at a rate of 0.5°C per second, with continuous measurement of the fluorescence, cooling to 60°C for 15 s. The relative expression levels of target genes were normalized by the maize β*-Tubulin 4* gene (Zm00001d013612) and relative to the sample at 0 hai by using the 2^−Δ*ΔCt*^ method. All qRT–PCR reactions were performed with three biological replicates for each sample.

### Oxylipin Profiling

Oxylipin profiling was conducted according to the description in previous studies (Wang et al., 2020; Ma et al., 2021), with some modifications. In brief, non-inoculated (control) and inoculated maize stem tissues were collected at 24 and 48 hai from the three genotypes, then immediately frozen with liquid nitrogen and stored in −80°C. Afterward, 100 mg of stem tissue was finely ground using liquid nitrogen and mixed with 500 μl extraction buffer (1-propanol/water/HCl [2:1:0.002 vol/vol/vol]) containing 10 μl of 5 μM isotopically-labeled internal standards. The samples were processed and injected into an API 3200 (Sciex, Framingham, MA, USA) LC-MS/MS using electrospray ionization in negative mode with multiple reactions mentoring (MRM). The chromatography was performed with an Ascentis Express C-18 Column (3 cm × 2.1 mm, 2.7 μm) (Sigma-Aldrich, St. Louis, MO, USA). The mobile phase was set at 400 ml per minute consisting of Solution A (0.02% acetic acid in water) and Solution B (0.02% acetic acid in acetonitrile) with a gradient consisting of (time in min—%B):0.5–10%, 1–20%, 21–70%, 24.6–100%, 24.8–10%, 29–stop. The peaks and retention times of oxylipins were then determined by comparing levels of endogenous metabolites to isotopically labeled standards from Sigma-Aldrich (St. Louis, MO, USA), Cayman Chemical (Ann Arbor, MI, USA), and Larodan AB (Solna, Sweden), with appropriate response factors (Muller and Munne-Bosch, 2011; Christensen et al., 2014). Using unsupervised principal component analysis (PCA), significantly regulated metabolites in each genotype and between three genotypes during the infection period were determined by one-way and two-way ANOVA analysis and were analyzed using MetaboAnalyst (version 5.0) (https://www.metaboanalyst.ca/) (Chong et al., 2019).

## Results

### *zmlox5-3* Mutant Is Shown to Be Susceptible to Gibberella Stalk Rot

**Figure 1:**
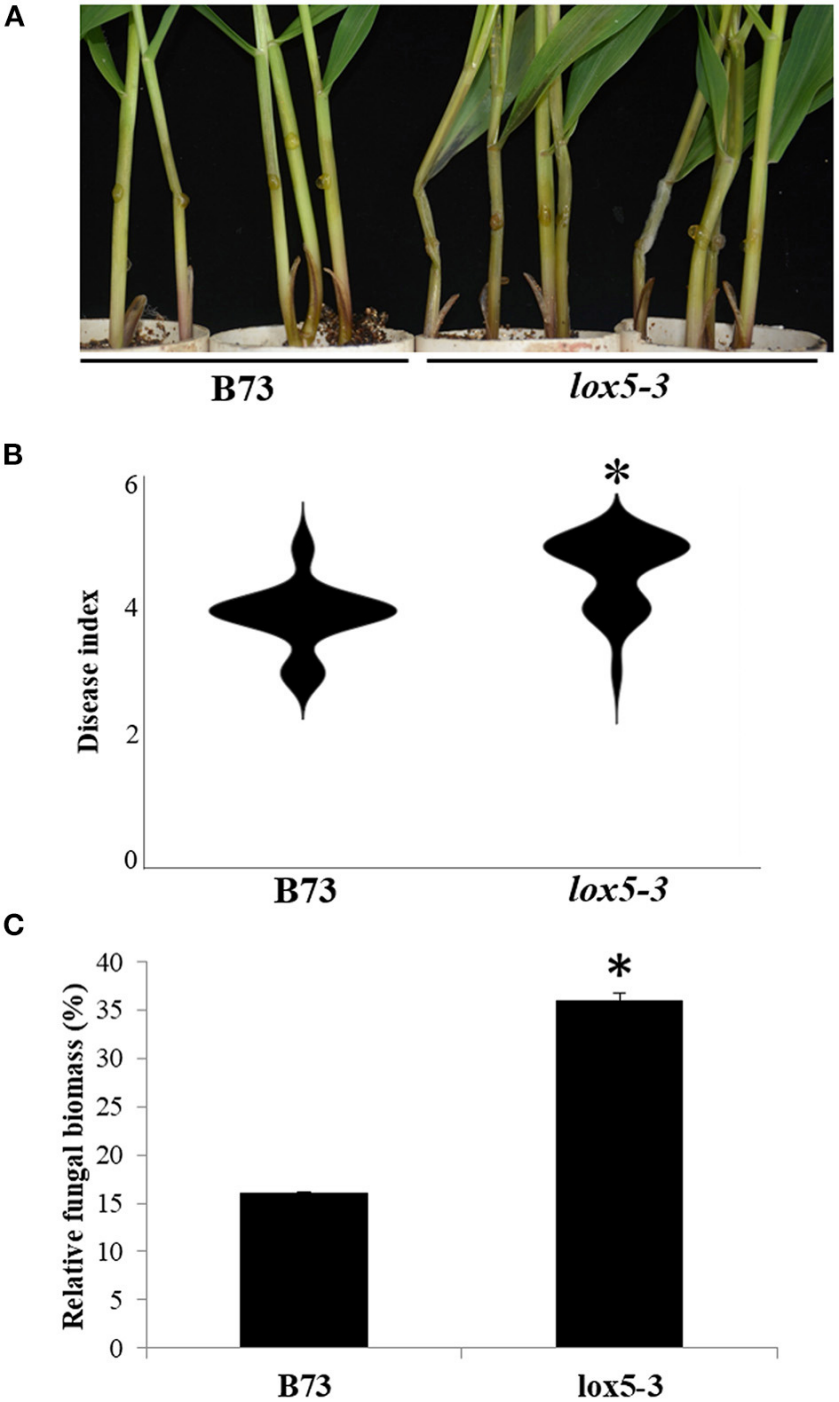
Phenotyping of *zmlox5-3* and B73 stems at 3 days post-infection with *F. graminearum*. **(A)** Representative images of B73 (left) and *lox5-3* (right) at 72 h after infection (hai) with *F. graminearum*. **(B)** Gibberella stalk rot (GSR) disease index of B73 and *zmlox5-3* at 72 hai. The disease was quantified using 20 seedlings per genotype. **(C)** Fungal biomass quantification of *F. graminearum* by qRT-PCR at 72 hai for B73 and *zmlox5-3*. The data were shown as the average of 10 plants per genotype. The experiments were replicated three times, with similar results obtained. Stars above the bars indicate the statistical significance at *p* < 0.05.

To elucidate the function of 9-oxylipins in GSR resistance of maize, we deployed *zmlox5-3*, a *Mutator*-insertional mutant of *ZmLOX5*, one of the 9-LOX gene (Park et al., 2010), and its near-isogenic WT, to the inbred line B73. Mutant *zmlox5-3* was previously reported to display increased susceptibility to *F. verticillioides* (Battilani et al., 2018). Figures 1A,B show that *zmlox5-3* was significantly more susceptible to *F. graminearum* exhibiting a disease index of 4.6, compared with 3.9 for B73. Moreover, qRT-PCR-based quantification of the fungal biomass indicated that *zmlox5-3* contained 34.7% more fungal RNA compared with B73 (Figure 1C). The increased disease severity in *zmlox5-3* suggests that *ZmLOX5* is essential for maize resistance to GSR.

### Transcriptomic Analysis of Maize in Response to *F. graminearum* Infection

To identify the key genes potentially regulated by the *ZmLOX5*-derived oxylipins and their involvement in maize–*F. graminearum* interaction, Illumina-based RNA-seq was conducted using stems collected from B73 and *zmlox5-3* seedlings at 12 and 24 h post-infection with *F. graminearum*. In total, the transcriptome sequencing of 12 libraries produced clean reads ranging from 33,576,369 to 19,936,838 (average 26,454,227) with a length of 150 bp among the samples after filtering out low-quality reads, adaptor reads, and high unknown base (N) reads. Using HISAT to map the reads against the B73 genome as a reference, more than 85 and 88% alignment rates were obtained in B73 and *zmlox5-3*, respectively (Supplementary Table 2). Pearson's correlation between biological replicates for each time point was higher than 77 and 85% in B73 and *zmlox5-3*, respectively, indicating good repeatability of the sequencing data (Supplementary Figure 1).

**Figure 2:**
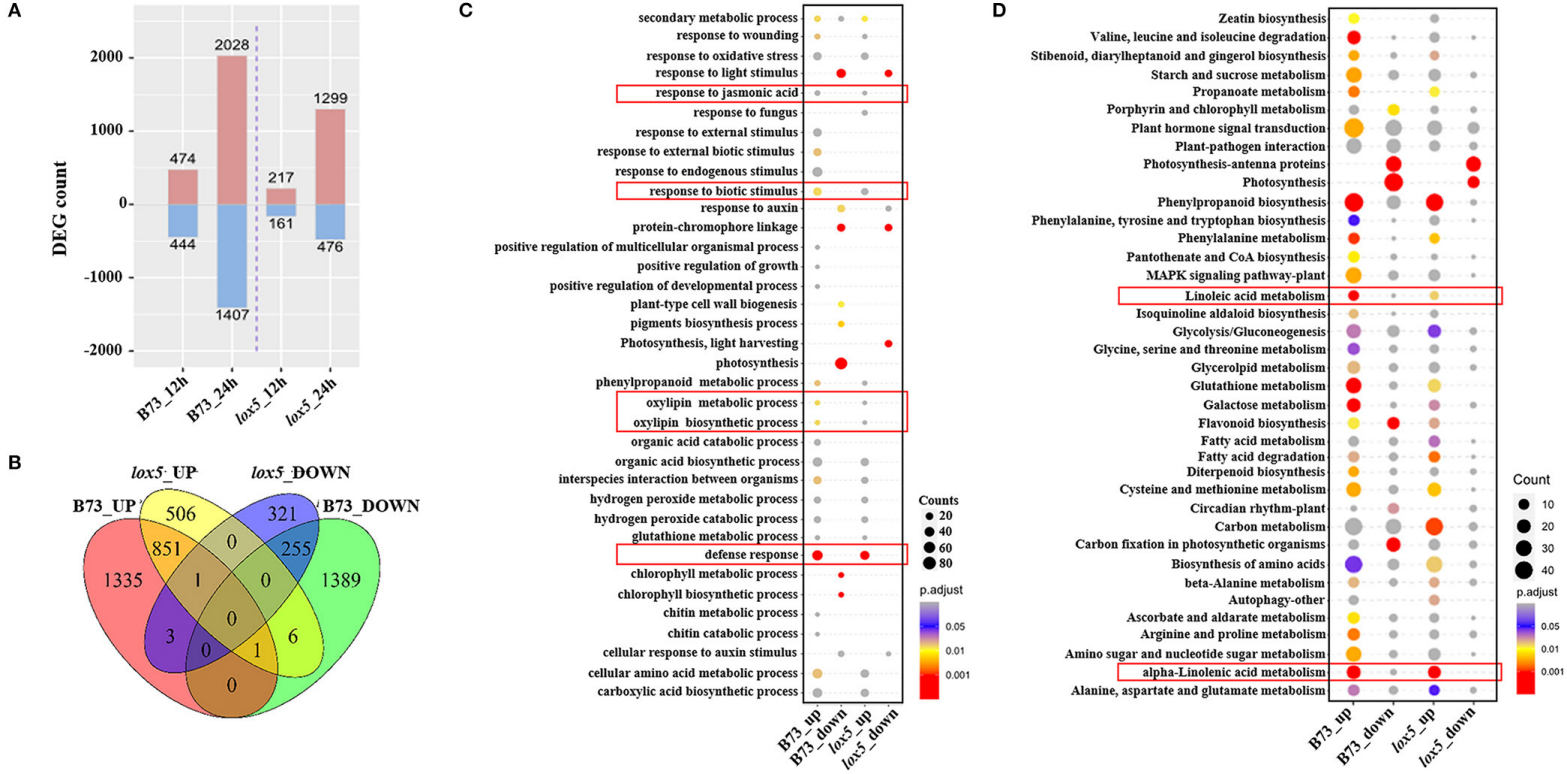
RNA-seq analysis of B73 and *zmlox5-3* upon *F. graminearum* infection. **(A)** Differentially expressed genes (DEGs) at 12 and 24 hai in B73 and *zmlox5-3*, respectively. DEGs were determined by comparing the expression of candidate genes in samples infected at 12 and 24 hai and that of mock samples, respectively. **(B)** The Venn diagram represented the specific and common DEGs between B73 and *zmlox5-3*. **(C)** GO term enrichment of up- and down-regulated DEGs significantly enriched in biological process (BP) in B73 and *zmlox5-3*. The terms associated with disease resistance are highlighted in the red rectangle. **(D)** Significantly enriched KEGG pathways of up- and down-regulated DEGs in B73 and *zmlox5-3*. The linoleic and alpha-linolenic acid metabolism were highlighted in the red rectangle. The size of the dot indicates the number of gene counts. The DEGs were selected with the criteria at adjusted *p* (*p*.adj) <0.05 shown in the colored sidebar.

Differentially expressed genes were then identified with a threshold of *p* < 0.05 and log2 Fold Change (FC) > 1. Compared with the controls, 474 and 2,028 up-regulated DEGs and 444 and 1,407 down-regulated DEGs were identified in B73. On the other hand, only 217 and 1,299 up-regulated DEGs and 161 and 476 down-regulated DEGs were recorded in *zmlox5-3* at 12 and 24 hai, respectively (Figure 2A). In total, 506 up-regulated and 321 down-regulated DEGs were uniquely expressed in *zmlox5-3*, whereas 1,335 up-regulated and 1,389 down-regulated DEGs were specifically identified in B73. There were 851 common up-regulated DEGs and 255 common down-regulated DEGs in B73 and *zmlox5-3* (Figure 2B).

To further determine the DEGs potentially relevant for GSR resistance or susceptibility, we conducted GO analysis for the up- and down-regulated genes in B73 and *zmlox5-3* (Figure 2C). It showed that GO terms of biological processes (BP) “chlorophyll metabolic process” and “chlorophyll biosynthetic process,” as well as “response to external biotic stimulus,” were only up-regulated in B73. Whereas, “photosynthesis” was the most down-regulated in B73 but not in *zmlox5-3*. On the contrary, “photosynthesis, light-harvesting” was only down-regulated in *zmlox5-3* but not in B73. Furthermore, multiple defense response-related processes, including “defense response,” “response to biotic stress,” “cellular amino acid metabolic process,” “phenylpropanoid metabolic process,” “response to jasmonic acid,” “oxylipin metabolic process,” and “oxylipin biosynthetic process” were all up-regulated in both genotypes.

To better understand the relative biological functions of DEGs in the two lines upon infection with *F. graminearum*, KEGG pathway analysis was conducted and enrichment of the pathways that were up-regulated or down-regulated was analyzed. As shown in Figure 2D, up-regulated DEGs were highly enriched in pathways such as “phenylpropanoid biosynthesis,” “phenylalanine metabolism,” “glutathione metabolism,” and “galactose metabolism” in both lines, whereas down-regulated DEGs were mainly enriched in the photosynthesis-related pathways, including “photosynthesis-antenna protein,” “photosynthesis,” and “carbon fixation pathways” (Figure 2D). Notably, “linoleic acid metabolism” and “α-linolenic acid metabolism” pathways were also found to be highly enriched for up-regulated DEGs in both lines (Figure 2D).

### Oxylipin Profiling of the Seedling Response to Infection With *F. graminearum*

**Figure 3:**
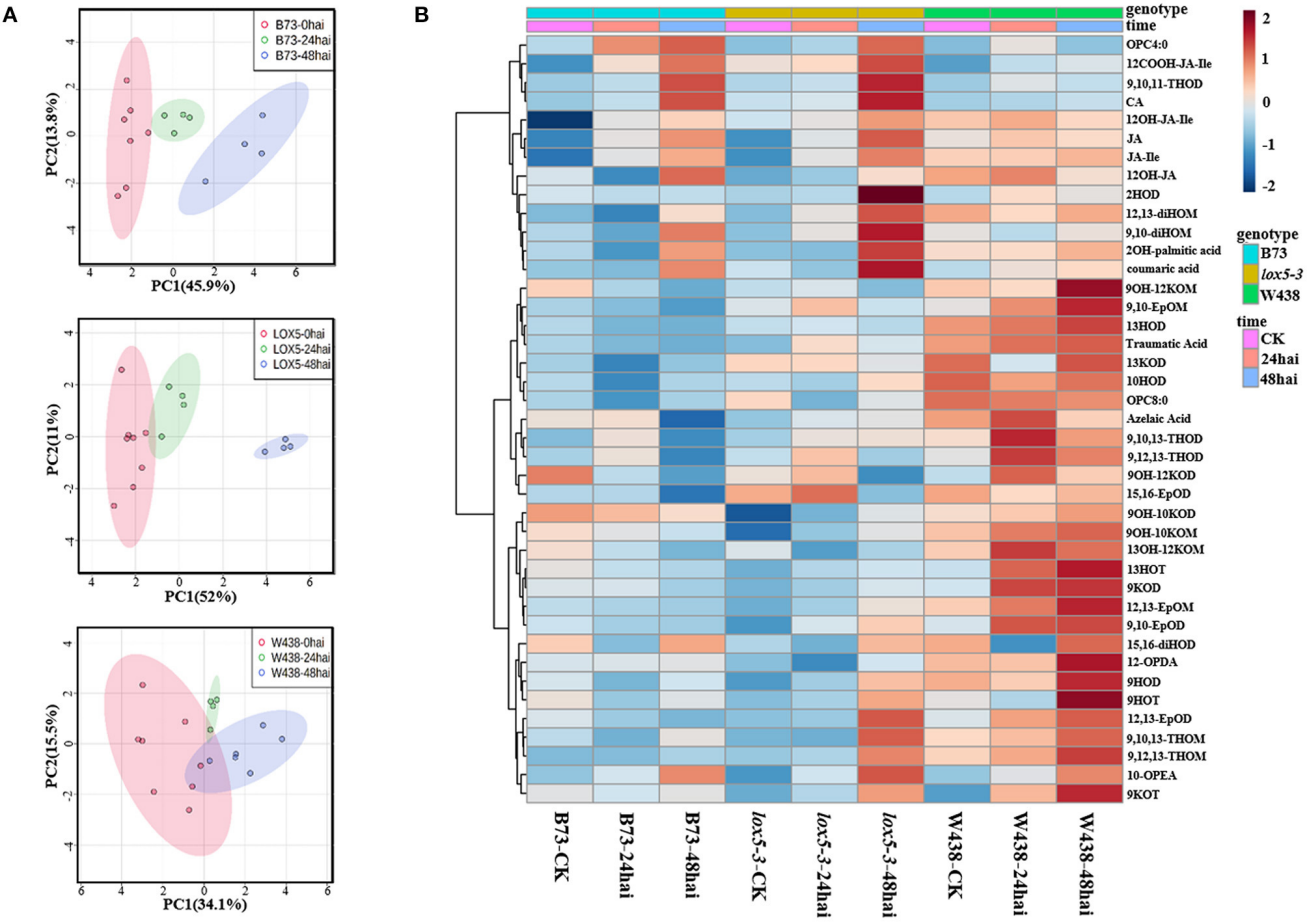
Oxylipins profiling in stem tissue of W438, B73, and *zmlox5-3* at 24 and 48 h and corresponding controls at 0 h post-infection with *F. graminearum*. **(A)** PCA score plot based on the metabolite profiling of W438, B73, and *lox5-3*. **(B)** Heatmap of significantly altered oxylipins between W438, B73, and *zmlox5-3* upon infection with *F. graminearum*. The sidebars on the left of the heatmap represent genotypes and time points, respectively. Statistical analysis was conducted by Two-way ANOVA (interaction *p* ≤ 0.01).

To identify oxylipins that may explain decreased resistance of *zmlox5-3* mutant, we performed oxylipin quantification in the mutant and B73 at different time points upon infection with *F. graminearum*. To hone-in a subset of oxylipins associated with GSR resistance, we included the GSR resistant inbred line W438 (Sun et al., 2021) in the panel subjected to oxylipin profiling. The principal component analysis showed a clear trend of separation between control and infected samples for all three lines. Interestingly, infected *zmlox5-3* at 48 hai was clearly separated from the uninfected control, compared with B73 and W438, suggesting that multiple oxylipins might be associated with GSR resistance or susceptibility (Figure 3A).

In total, we have measured 51 compounds. Two-way ANOVA analysis identified 42 metabolites that were significantly different at *p* ≤ 0.05 (Supplementary Table 3). The majority of these compounds showed distinct patterns in the three genotypes during the infection period (Figure 3B). Several 9-oxylipins were strongly induced in W438 at both 24 and 48 hai. For instance, the contents of 9OH-10KOM, 9,10-EpOM (9(*R*), 10(*S*)-Epoxy-12(*Z*)-octadecenoic acid), 9-HOT, and 9-HOD at 48 hai were 6.9, 3.8, 3.5, and 2.2 times higher in W438 than that in *lox5-3*, underlining the potential relevance of 9-oxylipins for defense against *F. graminearum*.

### Higher Jasmonates Levels Correlate With Increased Susceptibility to GSR

**Figure 4:**
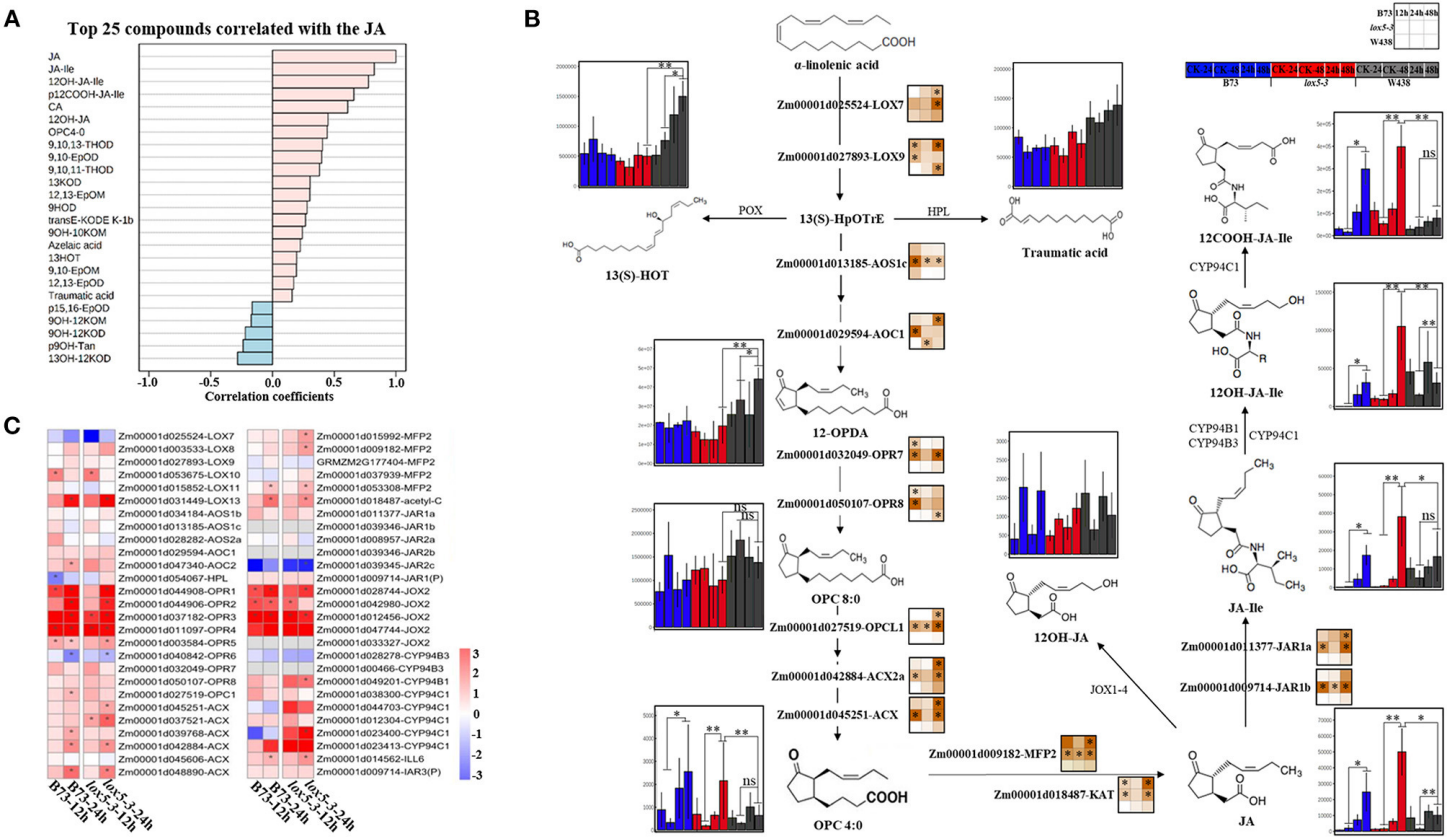
Integrated analysis of transcriptome and 13-oxylipins profiling of stem tissues of W438, B73, and *lox5-3* at 24 and 48 h post-infection with *F. graminearum*. The corresponding control was the samples collected at 0 hai. **(A)** Correlation coefficient analysis of oxylipins associated with JA between W438, B73, and *zmlox5-3* upon *F. graminearum* infection. **(B)** Diagram of main oxylipins and expression of related genes involved in JA biosynthesis pathway. The change of oxylipin contents was indicated as a box plot at the side of the compound name, and the heat map at the side of the gene name represented the expression levels of selected genes. The stars indicate statistical significance at *p* < 0.05. **(C)** Expression pattern of genes involved in the 13-LOX pathway in B73 and *zmlox5-3* at 12 and 24 hai. The different colors represent the log2 fold-change of each gene at different time points, and stars indicate the statistical significance at *p* < 0.05.

In contrast to 9-oxylipins, most JA derivatives, including OPC4:0 [3-oxo-2-(2-pentenyl) cyclopentane-1-butanoic acid], JA, JA-Ile, 12OH-JA, 12COOH-JA-Ile, and 12OH-JA-Ile, were significantly induced in *lox5-3* at 48 hai and to a lesser extent in B73. Remarkably, the levels of jasmonates remained unchanged in the resistant inbred W438. To better understand the potential interaction between JA and other oxylipins, we examined metabolites that were related to JA. All jasmonates showed a positive correlation as expected. On the contrary, 15,16-EpOD, 9OH-12KOM, 9OH-12KOD, 9OH-Tan, and 13OH-12KOD negatively correlated with JA (Figure 4A), suggesting that these oxylipins may be antagonistic to JAs.

To understand how JAs and other 13-oxylipins are impacted by *ZmLOX5* mutation at the transcriptional and metabolic levels, we conducted the joint analysis of metabolites and gene expression levels of 13-oxylipin pathways in WT and *zmlox5-3* mutant. While the majority of the compounds in the JA biosynthetic pathways, including OPC4:0, JA, and JA derivatives, especially JA-Ile, were produced at a relatively lower level in the infected W438, these metabolites were significantly induced in *lox5-3* (Figure 4B). Surprisingly, the concentration of the compounds upstream of OPC:0, such as 12-OPDA and OPC8:0, were maintained at higher levels in W438 but not in *zmlox5-3*. Among those compounds, JA-Ile levels increased ~14.8-fold and 43.5-fold in *zmlox5-3* at 24 and 48 hai, and 22.7-fold to 43.2-fold in B73, respectively, whereas only 0.9-fold to 2.7-fold increases were observed in W438 at 24 and 48 hai, respectively (Supplementary Table 4).

In line with the results of oxylipin profiling, the key genes encoding proteins converting JA to JA-Ile are *Jasmonate resistant 1* (*JAR1a*, Zm00001d011377) and *JAR1b* (Zm00001d009714) and the two putative JA-Ile conjugating enzymes in maize (Borrego and Kolomiets, 2016), which were highly expressed at most time points in *zmlox5-3* but induced only at 48 hai in B73. Moreover, two genes encoding proteins metabolizing OPC4:0 to JA, which are *glyoxysomal fatty acid beta-oxidation multifunctional protein MFP-a* (*MFP2*, Zm00001d009182) and *acetyl-CoA acyltransferase* (*KAT*, Zm00001d018487), were both up-regulated in B73 and *lox5-3*. However, all these genes were not induced throughout all time points in W438 (Figure 4B), supporting the notion that increased JA is associated with increased susceptibility to GSR.

Surprisingly, the precursor compounds of the JA biosynthetic pathway, such as 12-OPDA and OPC8:0, accumulated to higher levels in W438, compared with the susceptible genotypes (Figure 4B), which may be due to reduced conversion of these precursors to JA in W438. Interestingly, several key genes encoding enzymes catalyzing the formation of JA precursors, including *LOX9* (Zm00001d027893), *AOC1* (Zm00001d029594), and *OPR8* (Zm00001d050107), were up-regulated earlier at 12 hai in *zmlox5-3*, but at later time points at 24 and 48 hai in B73 and W438, respectively. Moreover, most of the genes engaged in three-step β-oxidation, including *OPC-8:0 CoA ligase 1* (*OPCL1*, Zm00001d027519), *Acyl-coenzyme A oxidase* (*ACX*, Zm00001d045251), *MFP2*, and *KAT*, were highly expressed in *zmlox5-3* and to a lesser extent in B73, but not in W438 (Figure 4B). Taken together, these results suggest that JA biosynthesis pathway genes downstream of 12-OPDA were induced in *zmlox5-3* and B73 but not in resistant W438. This again points to JA-Ile as a potential susceptibility factor promoting virulence of this pathogen, a scenario similar to a recent study showing that JAs contribute to susceptibility toward another hemibiotrophic maize pathogen, *Colletotrichum graminicola* (Gorman et al., 2020).

### Accumulation of 9-Oxylipins Is Associated With Reduced Susceptibility

**Figure 5:**
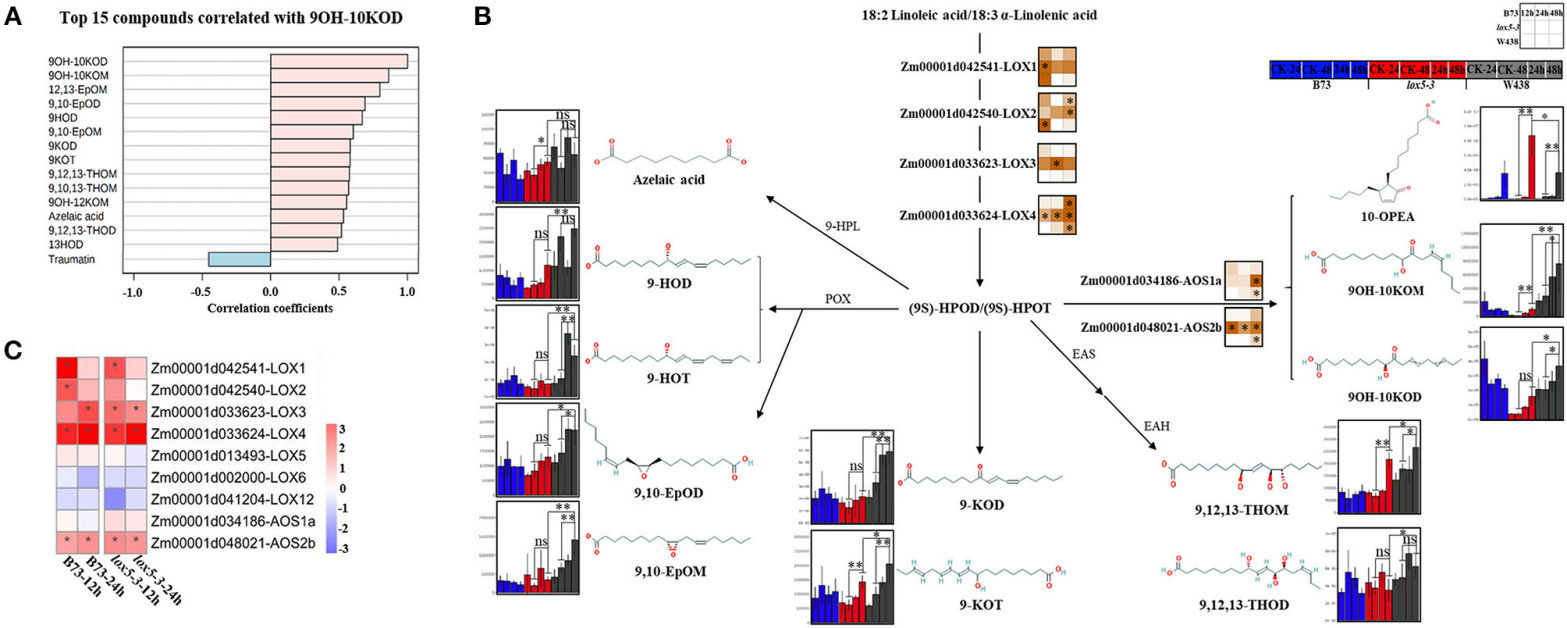
Integrated analysis of transcriptome and 9-oxylipins profiling of stem tissues of W438, B73, and *lox5-3* at 24 and 48 h post-infection with *F. graminearum*. The corresponding control was the samples collected at 0 hai. **(A)** Correlation coefficient analysis of oxylipins associated with 9OH-10KOD between W438, B73, and *lox5-3* upon *F. graminearum* infection. **(B)** Diagram of main oxylipins and expression of related genes involved in the 9-LOX pathway. The change of oxylipin contents is indicated as a box plot at the side of the compound name, and the heat map at the side of the gene name represented the expression levels of selected genes. The stars indicate the statistical significance at *p* < 0.05. **(C)** Expression pattern of genes involved in the 9-LOX pathway in B73 and *lox5-3* at 12 and 24 hai. Different colors represent log2 fold-change of each gene at different time points, and stars indicate the statistical significance at *p* < 0.05.

Given that ZmLOX5 is a 9-LOX, we investigated whether 9-oxylipins could be associated with GSR resistance. Quantification of 9-oxylipins showed that an α-ketol, 10-oxo-9-hydroxy-12(*Z*) and 15(*Z*)-octadecadienoic acid (9OH-10KOD), which is recently identified as a mobile ISR signal (Wang et al., 2020), was the most significantly induced compound in W438 (Supplementary Table 3). Therefore, we further examined which other oxylipins correlated with 9OH-10KOD (Figure 5A). It was clearly shown that 11 out of 14 oxylipins identified in this study produced through the 9-LOX pathway highly correlated with 9OH-10KOD, whereas traumatin was the only compound negatively correlated with 9OH-10KOD (Figure 5A). These 9-oxylipins, which are mainly produced by 9-POX, 9-AOS, EAS, EAH, 9-HPL, and 9-LOX branch pathways, were significantly induced in W438 but displayed moderate or no change in B73, and much lower basal levels in the *lox5-3* mutant (Figure 5B). Moreover, the contents of the two ketols, 9OH-10KOM and 9OH-10KOD, were 6.87- and 2.45-fold higher in W438 than in *zmlox5-3* at 48 hai (Figure 5B). Similarly, 9,10-EpoM and 9-HOT levels were 3.75- and 3.45-fold higher in W438 than in *zmlox5-3* at 48 hai (Supplementary Table 4).

Intriguingly, the so-called “death acid” 10-oxo-11-phytodienoic acid (10-OPEA) accumulated to higher levels in *zmlox5-3* than in B73 or W438 at 48 hai. The analysis of qRT-PCR showed that the expression levels of the 9-LOXs and putative death acid producers, which are *ZmLOX1* (Zm00001d042541), *ZmLOX2* (Zm00001d042540), *ZmLOX3* (Zm00001d033623), and *ZmLOX4* (Zm00001d033624), were higher in *zmlox5-3*. Whereas, in W438, *ZmLOX1* and *ZmLOX2* were strongly induced only at the earlier time point at 12 hai and *ZmLOX4* was highly expressed at 48 hai (Figure 5B). Similar to 9-LOXs, putative ketol- and death acid-producing *AOS2b* (Zm00001d048021) also displayed higher expression levels in the *zmlox5-3* mutant (Figure 5B). Together, these results suggest that the cell death process mediated by “death acids” could be associated with the increased susceptibility of *zmlox5-3* observed.

## Discussion

Compared with JAs, the role of a large number of 9-oxylipins generated *via* the 9-LOX pathways in plant resistance to pathogens remains less explored. Among the well-studied maize 9-LOXs, *zmlox3* mutant displayed enhanced resistance to multiple pathogens, including *Cochliobolus heterostrophus, F. verticillioides, C. graminicola*, and *Ustilago maydis*. This reveals that this specific gene is a susceptibility factor in response to these pathogens (Gao et al., 2007; Battilani et al., 2018; Pathi et al., 2020). On the contrary, the other 9-LOX genes, *ZmLOX4, ZmLOX5*, and *ZmLOX12* were reported to be required for defense against *F. verticillioides* in maize, as *lox4* and *lox5* mutants were susceptible to *F. verticillioides*, accompanied by decreased levels of JAs (Park, 2012; Christensen et al., 2014; Battilani et al., 2018, Lanubile et al., 2021). The present study showed that ZmLOX5 is involved in defense against GSR and revealed an opposite role of 9-oxylipins and JAs in response to *F. graminearum*. Similar to these findings, Wang et al. (2020) have also demonstrated contrasting roles of 9-LOX-derived ketols and JA in maize resistance to anthracnose leaf blight pathogen, *C. graminicola*. In that study, while the treatment of maize with an α-ketol, 9,10-KODA resulted in increased resistance to *C. graminicola*, JA facilitated virulence of this pathogen. The negative role of JA in defense against *C. graminicola* was explained by its antagonistic interaction with SA as a JA-deficient mutant of maize was more resistant to this pathogen and accumulated increased levels of SA (Gorman et al., 2020). It is important to note, that both *F. graminearum* and *C. graminicola* are well-established hemibiotrophic pathogens, the defense against which primarily relies on SA signaling. Thus, it appears that individual molecular species of oxylipins exert their function in disease resistance or susceptibility in a pathogen species-dependent manner.

### Increased JA Correlates With Increased Susceptibility to *F. graminearum*

Oxylipin profiling performed in this study showed that the majority of jasmonates were highly induced in *zmlox5-3* compared with WT in response to *F. graminearum* infection, far exceeding the levels found in the resistant line W438 (Figure 4B). This suggests a positive correlation of JAs with enhanced GSR susceptibility in *zmlox5* mutant. Increased JA synthesis in the mutant was further supported by increased expression of both JA biosynthesis genes and the genes regulated by JA in the *zmlox5-3* mutant. These results also revealed that the 9-oxylipins produced by ZmLOX5 have a profound suppressive effect on the biosynthesis of JAs in response to *F. graminearum*. A strong antagonism between JA, 13-oxylipins, and 9-oxylipins has been recently demonstrated for wound responses of maize JA-deficient *opr7opr8* double mutant and a mutant in the major 13-LOX gene, *ZmLOX10* (He et al., 2020). That study showed that deletion of 13-oxylipins resulted in overproduction of a number of wound-induced 9-oxylipins.

Jasmonic acids and their derivatives have been repeatedly mentioned for their roles in defense against necrotrophic pathogens in various plant species (Howe, 2001; Stintzi et al., 2001; Wasternack and Hause, 2013; Borrego and Kolomiets, 2016; Wasternack and Feussner, 2018; Deboever et al., 2020). However, multiple lines of evidence in multiple plant species suggest that JA promotes disease progression by biotrophic and hemibiotrophic pathogens due to the well-characterized antagonism between JA and SA signaling (Glazebrook, 2005). One of the strongest illustrations of JA signaling serving to promote pathogen virulence is the discovery that some pathovars of *Pseudomonas suringae* take advantage of JA-SA antagonism by producing coronatine, a structural and functional mimic of JA-Ile, to induce host JA signaling, which in turn results in the down-regulation of SA-mediated defenses (Robert-Seilaniantz et al., 2011; Pieterse et al., 2012). In the case of this study, due to the hemibiotrophic lifestyle of *F. graminearum*, overproduction of JAs is beneficial for *F. graminearum* to promote disease progression at the necrotrophic phase, e.g., after 48 h post-infection. We hypothesize that JA promotes disease progression at the necrotrophic phase of infection by its well-known capacity to induce cell death processes in diverse plant species including maize (Mur et al., 2006; Yan et al., 2012). The finding that JA promotes susceptibility to GSR is supported by the recent study showing the increased resistance against *F. graminearum* in the *COI1* and JA-deficient mutants of maize (Ma et al., 2021), and by the previous study in Arabidopsis that *coi1* mutant is more resistant to wilt disease caused by *F. oxysporum* (Thatcher et al., 2009). The results in the present study, together with previous findings, support a strong negative relationship between JA pathways and resistance to diseases caused by *Fusarium* spp.

In this study, the levels of bioactive JA-Ile in the susceptible lines, *zmlox5-3* and B73, were excessively high in comparison to the resistant line W438 (Figure 4B). This might be due to the limitation of over-synthesis of JA-Ile in resistant line (Staswick and Tiryaki, 2004; Stitz et al., 2011; Caarls et al., 2017; Smirnova et al., 2017). Another reason for increased JAs in *zmlox5-3* might be the constant catalysis via β-oxidation, the process that appears to be exerted at a much lower level in W438, as evident from both transcript levels of β-oxidation genes and actual JA precursors' contents.

A recent study showed that upon activation of induced systemic resistance (ISR) by *Trichoderma virens* in B73, 12-OPDA levels increased, while JA biosynthetic genes downstream of 12-OPDA, such as *ACX, MFP* and *KAT*, and JA responsive genes were down-regulated. In here, as well as in the application of exogenous JA and JA-Ile, it resulted in increased susceptibility to *C. graminicola* (Wang et al., 2020). These results, together with the present data of this study, suggest that individual steps in JA biosynthetic pathways seem to be tightly controlled in a pathogen-dependent manner to produce diverse intermediate products, whose functions might be distinct from those of the final functional jasmonates JA-Ile. Additionally, pathogens might also produce JA, JA-Ile, and JA-Ile analogs to facilitate their pathogenicity (Gao and Kolomiets, 2009; Brodhun et al., 2013; Cole et al., 2014; Geng et al., 2014; Patkar et al., 2015; Gimenez-Ibanez et al., 2016; Deboever et al., 2020). Thus, it is reasonable to propose that the bioactive hormone JA-Ile is likely regulated by ZmLOX5 in a resistant line to avoid its overproduction. Although the exact mechanisms behind *ZmLOX5-*mediated suppression of jasmonates synthesis are not fully understood, our results provided genetic evidence that this gene and its products play a vital role in defense against GSR via negative regulation of JA and JA-Ile biosynthesis.

### ZmLOX5-Derived 9-Oxylipins Contribute to Resistance to GSR

It has been shown that 9-oxylipins possessed strong activity in defense against various pathogens in several plant species (Christensen et al., 2015; Deboever et al., 2020). For instance, 9-hydroxy-10*E*,12*Z*-octadecadienoic acid (9-HOD) was found to be 20-fold higher in WT than in the susceptible mutant of *Nicotiana attenuata* upon bacterial infection (Schuck et al., 2014). Moreover, a greater 9-KOT level was shown to correlate with increased resistance (Vicente et al., 2012), while (9*S*, 10*E*, 12*Z*, 15*Z*)-9-hydroxy-10,12,15-octadecatrienoic acid (9-HOT) was suggested to participate in defensive responses and cell wall modification during the reaction to hemibiotrophic bacteria in Arabidopsis (Vellosillo et al., 2013). Similarly, in the present study, the contents of multiple 9-oxylipins, including 9OH-10KOM, 9OH-10KOD, 9-HOT, 9-HOD, 9-KOD, and 9-KOT, were much higher in the resistant line W438 than in susceptible *zmlox5-3*, emphasizing the important role of 9-oxylipins in resistance to GSR.

Another 9-oxylipin, 10-OPEA, has been reported to act as a phytoalexin and an inducer of plant programmed cell death (PCD) to suppress some fungal pathogens (Christensen et al., 2015; He et al., 2019). However, our results did not support such a role for this molecule in maize interaction with *F. graminearum*. We showed that this oxylipin and the putative biosynthetic gene encoding a 9-AOS, *AOS2b*, were more strongly induced in *zmlox5-3* compared with B73 and W438 (Figure 5B). One possible reason behind such opposite roles of 10-OPEA is that high 10-OPEA content was related with PCD caused by infection of necrotrophic pathogen *Cochliobolus heterostrophus* in the previous study, despite the minor effect of 10-OPEA on fungal growth *in vitro* (Christensen et al., 2015), whereas *F. graminearum* possess hemibiotrophic lifestyle. This is reminiscent of the widely published contrasting roles of JA-Ile in resistance to necrotrophs, but in susceptibility to hemibiotrophs. However, further experiments will be needed to show whether higher levels of 10-OPEA are indeed associated with enhanced susceptibility of *zmlox5-3* mutants.

In summary, this study has shown that *zmlox5-3* mutant accumulates higher amounts of diverse jasmonates together with greater expression levels of JAs, but lower levels of 9-oxylipins and reduced gene expression of *9-LOXs*. On the contrary, lower amounts of JAs but higher levels of multiple 9-oxylipins were found in the resistant line. These findings indicate that 9-oxylipins and JAs pathways might antagonize each other and that *ZmLOX5*-regulated 9-oxylipins contribute to resistance, whereas JAs seem to play a negative role in maize defense against GSR.

## Data Availability Statement

The data presented in the study are deposited in the NCBI GEO database, accession number GSE174508.

## Author Contributions

XG and MK: conceptualization and resources. QW, P-CH, and YW: data curation. QW, YS, FW, and P-CH: formal analysis. XG: funding acquisition, project administration, and supervision. QW, YS, FW, P-CH, XR, LM, and XL: investigation. QW, YS, P-CH, and XR: methodology. QW and YS: validation. QW and XG: writing-original draft preparation. QW, XG, and MK: writing-review and editing. All authors read and approved the final manuscript.

## Funding

This work was supported financially by the grants from The National Key Research and Development Program of China (No. 2016YFD0101002), National Natural Science Foundation of China (Nos. 31671702 and 31471508), and Jiangsu Collaborative Innovation Center for Modern Crop Production (JCIC-MCP).

## Conflict of Interest

The authors declare that the research was conducted in the absence of any commercial or financial relationships that could be construed as a potential conflict of interest.

## Publisher's Note

All claims expressed in this article are solely those of the authors and do not necessarily represent those of their affiliated organizations, or those of the publisher, the editors and the reviewers. Any product that may be evaluated in this article, or claim that may be made by its manufacturer, is not guaranteed or endorsed by the publisher.

## Supplementary Material

Supplementary Figure 1 RNA-seq data correlation of different replicates from B73 and *zmlox5-3* at 0, 12 and 24 h post-*F. graminearum* inoculation.

Supplementary Figure 2 Gene Ontology (GO) term enrichment of up- and down-regulated differentially expressed genes (DEGs) significantly enriched in **(A)** Cellular component (CC) and **(B)** Molecular function (MF) in B73 and *zmlox5-3*.

Supplementary Table 1 List of primers for qRT-PCR used in this study.

Supplementary Table 2 Statistics of all samples mapped to the B73 reference genome.

Supplementary Table 3 Two-way ANOVA (genotype × time) statistical analysis of oxylipins identified in this study.

Supplementary Table 4 Content fold change of oxylipins identified in B73, *lox5-3*, and W438 at 24 and 48 hai. The fold changes of oxylipins were calculated based on the contents in infected samples over that in control samples at 24 and 48 hai, respectively.
