# Supplementary figures and images for: Transcriptome and Oxylipin Profiling Joint Analysis Reveals Opposite Roles of 9-Oxylipins and Jasmonic Acid in Maize Resistance to Gibberella Stalk Rot
Source: Front Plant Sci. 2021 Sep 7;12:699146. doi: 10.3389/fpls.2021.699146 (PMC8454893; doi:10.3389/fpls.2021.699146)

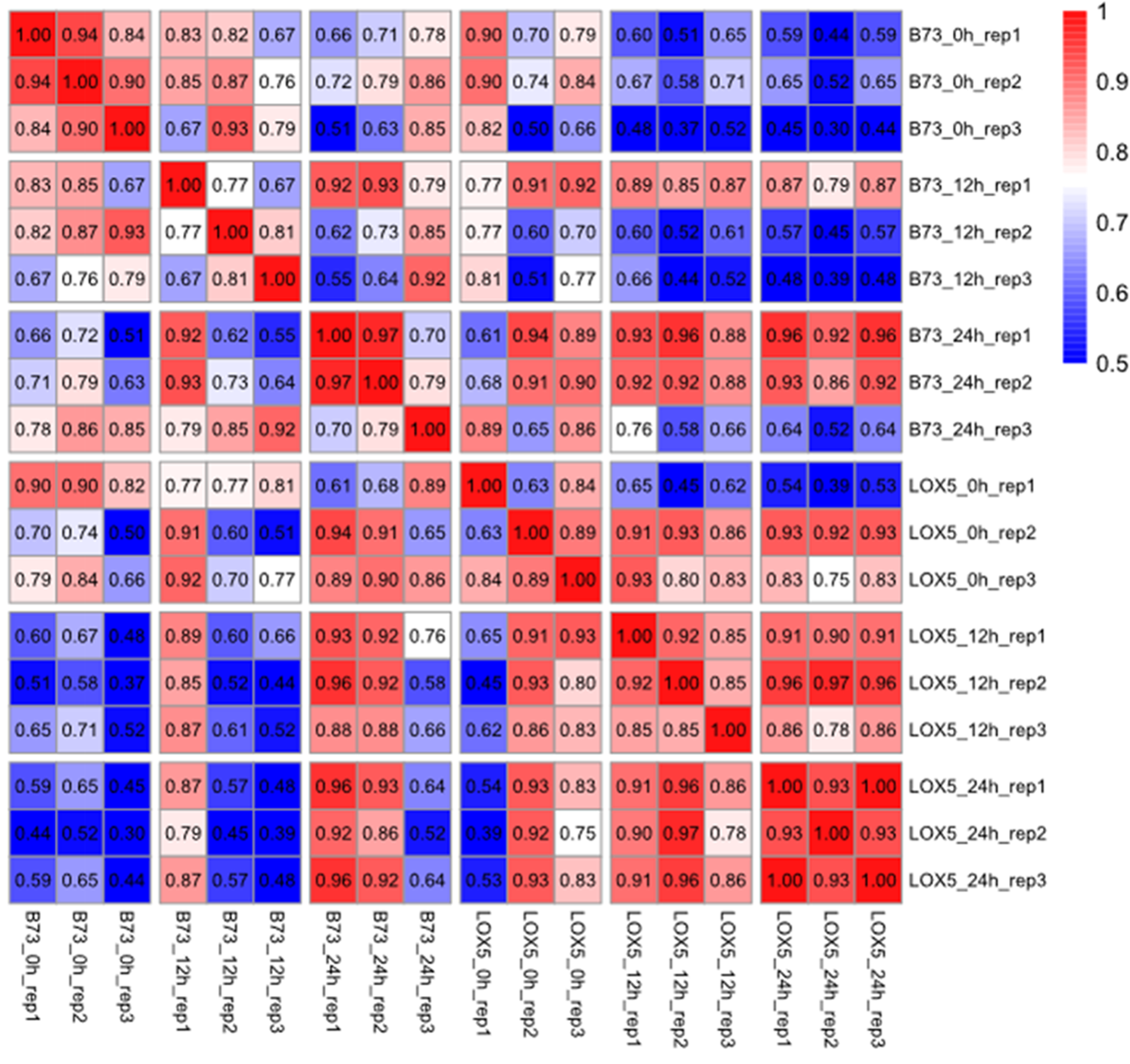

Supplement: Supplementary Figure 1 — RNA-seq data correlation of different replicates from B73 and zmlox5-3 at 0, 12 and 24 h post-F. graminearum inoculation. [file Image_1.TIF]

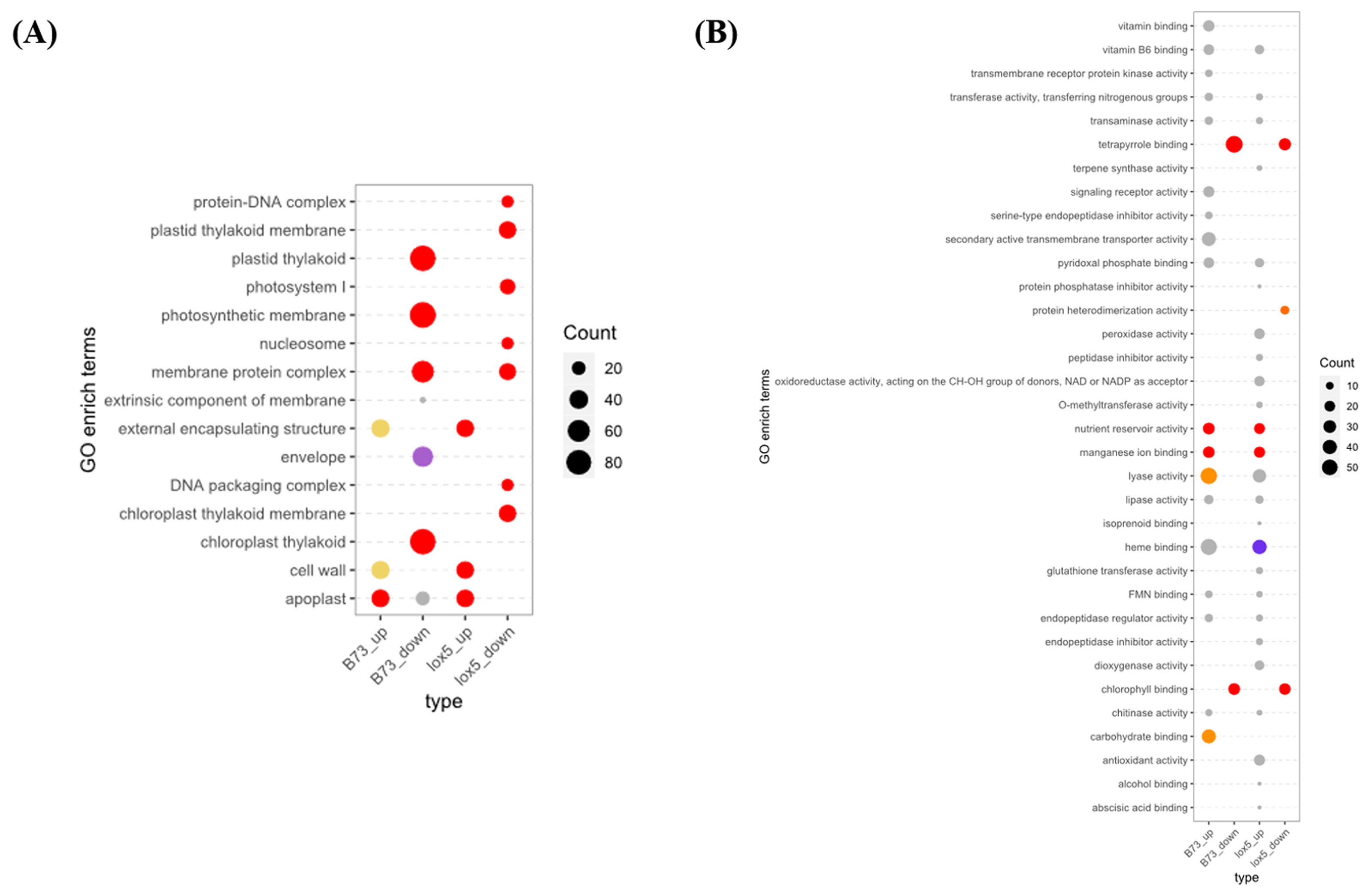

Supplement: Supplementary Figure 2 — Gene Ontology (GO) term enrichment of up- and down-regulated differentially expressed genes (DEGs) significantly enriched in (A) Cellular component (CC) and (B) Molecular function (MF) in B73 and zmlox5-3. [file Image_2.TIF]
