# Supplementary material for: Transcriptome and Oxylipin Profiling Joint Analysis Reveals Opposite Roles of 9-Oxylipins and Jasmonic Acid in Maize Resistance to Gibberella Stalk Rot
Source: Front Plant Sci. 2021 Sep 7;12:699146. doi: 10.3389/fpls.2021.699146 (PMC8454893; doi:10.3389/fpls.2021.699146)
Supplement: Supplementary Table 1 — List of primers for qRT-PCR used in this study. [file Table_1.DOCX]

**Supplemental Table 1. List of the genes and the primer sequences for qRT-PCR used in this study.**

| Gene | Forward Primer | Reverse Primer |
| --- | --- | --- |
| Fg Tublin | GGTCTCGACAGCAATGGTGTT | GCTTGTGTTTTTCGTGGCAGT |
| Zm00001d013612-β-Tubulin 4 | CTACCTCACGGCATCTGCTATGT | GTCACACACACTCGACTTCACG |
| Zm00001d042541-LOX1 | TCTGTCTGAGCTGAGGACGTA | CACAAAGTAACTTCATTATTGAGGA |
| Zm00001d042540-LOX2 | TTCCATCTGATTCGATCGAG | CACATTATTATTGGGAAACCAAC |
| Zm00001d033623-LOX3 | TACCACTACCACCCCAGGAGT | AGCACTGCGAAACGACTAGAA |
| Zm00001d033624-LOX4 | TGAGCGGATGGTTTGTAGAT | ATTATCCAGACGTGGCTCCT |
| Zm00001d025524-LOX7 | GCGAATGCGAGCTGTTTTGA | CGAACGATCTCCCCAGTAGC |
| Zm00001d027893-LOX9 | TGAGTGCATCGTTCGTTGT | TCAATCCTCATTCTTGGCAG |
| Zm00001d034186-AOS1a | GTACTGACACGCTCGCCTTA | ACAGCGAGTGACGTGTGCA |
| Zm00001d034184-AOS2b | ACAAGGTGGAGAAGAAGGAC | GTCGTTGAGCTTGTTGAACT |
| Zm00001d013185-AOS1c | GAACTGAGGGAGACATGCACA | GAGGTCATTTTAGGTTCTGATCATT |
| Zm00001d029594-AOC1 | ACGCATCCCTCGACAACTAC | AGCCAAACTACGGCTACGAG |
| Zm00001d011377-JAR1a | TCGAGGAAGACCCAGACCAT | ACCAGTACTTGGTGCGTAGC |
| Zm00001d009714-JAR1b | CGCCGCTCTCGGTGATAAAT | AGCGGGATGCAGGATTTGTA |
| Zm00001d027519-OPC1 | GGGGGCCATACGTCATGAAA | ACGTAGGCCATTGGGAACTG |
| Zm00001d045251-ACX | CGTGGAAGGACCCTCTGAAC | CGGAGGTCGCGTGAGATTTT |
| Zm00001d042884ACX2a | AGTGGTTGGTTGCCACAGAA | TGTGTTGCATGATTGGCAGC |
| Zm00001d018487-KAT | CGGTGTGTGGCTACTCTTCT | GTCCCTCACGTTGGAGAGTC |
| Zm00001d009182-MFP2 | TCTAGGAGCATGGCAGGAGT | CATGGATGTACTTTGCGCCG |
| Zm00001d032049-OPR7 | CTCCGCTCCCCA TTTCCCTCTCC | CAATCGCGGCATTACCCAGATGT |
| Zm00001d050107-OPR8 | TGGATAAAGGTGGTTGATGCTG | GACTGCTGATTGCTGTGCAAAT |
