# Supplementary material for: Transcriptome and Oxylipin Profiling Joint Analysis Reveals Opposite Roles of 9-Oxylipins and Jasmonic Acid in Maize Resistance to Gibberella Stalk Rot
Source: Front Plant Sci. 2021 Sep 7;12:699146. doi: 10.3389/fpls.2021.699146 (PMC8454893; doi:10.3389/fpls.2021.699146)
Supplement: Supplementary Table 2 — Statistics of all samples mapped to the B73 reference genome. [file Table_2.DOCX]

**Supplementary Table 2. Statistics of all samples mapped to B73 reference genome.**

| Genotypes | Time points | Biological replicates | All reads | Mapped reads | Unmapped reads | Mapping rates |
| --- | --- | --- | --- | --- | --- | --- |
| B73 | Control | 1 | 33,576,369 | 31481204 | 2,095,165 | 93.76% |
|  |  | 2 | 18,334,464 | 17,199,561 | 1,134,903 | 93.81% |
|  |  | 3 | 33,079,558 | 29,874,149 | 3,205,409 | 90.31% |
|  | 12hai | 1 | 21,552,302 | 20,332,442 | 1,219,860 | 94.34% |
|  |  | 2 | 19,936,838 | 18,160,466 | 1,776,372 | 91.09% |
|  |  | 3 | 22,237,049 | 19,235,048 | 3,002,001 | 86.50% |
|  | 24hai | 1 | 28,249,036 | 26,441,098 | 1,807,938 | 93.60% |
|  |  | 2 | 31,531,168 | 29,002,369 | 2,528,799 | 91.98% |
|  |  | 3 | 31,326,673 | 26,737,316 | 4,589,357 | 85.35% |
| lox5-3 | Control | 1 | 30,850,089 | 27,552,215 | 3,297,874 | 89.31% |
|  |  | 2 | 26,556,999 | 25,114,954 | 1,442,045 | 94.57% |
|  |  | 3 | 22,909,550 | 20,577,358 | 2,332,192 | 89.82% |
|  | 12hai | 1 | 26,612,180 | 23,506,539 | 3,105,641 | 88.33% |
|  |  | 2 | 23,131,543 | 21,931,016 | 1,200,527 | 94.81% |
|  |  | 3 | 20,650,205 | 18,587,250 | 2,062,955 | 90.01% |
|  | 24hai | 1 | 28,324,028 | 25,641,743 | 2,682,285 | 90.53% |
|  |  | 2 | 31,618,214 | 29,676,856 | 1,941,358 | 93.86% |
|  |  | 3 | 25,699,828 | 23,350,864 | 2,348,964 | 90.86% |

Notes: The above are the alignment rates of the transcriptome data of 0, 12 and 24 hours after inoculation (hai) from B73, *lox5-3* corresponding to the maize reference genome. Each time point contains three replicates.
