# Supplementary material for: Transcriptome and Oxylipin Profiling Joint Analysis Reveals Opposite Roles of 9-Oxylipins and Jasmonic Acid in Maize Resistance to Gibberella Stalk Rot
Source: Front Plant Sci. 2021 Sep 7;12:699146. doi: 10.3389/fpls.2021.699146 (PMC8454893; doi:10.3389/fpls.2021.699146)
Supplement: Supplementary Table 3 — Two-way ANOVA (genotype × time) statistical analysis of oxylipins identified in this study. [file Table_3.DOCX]

| **Oxylipins** | **genotype**  **(*p*. value)** | **Time**  **(*p*. value)** | **Interaction**  **(*p*. value)** |
| --- | --- | --- | --- |
| 12OH-JA-Ile | 2.01E-06 | 1.12E-09 | 1.02E-09 |
| CA | 2.33E-05 | 7.57E-10 | 7.33E-06 |
| 9OH-10KOD | 2.01E-06 | 0.0014864 | 9.68E-06 |
| JA-Ile | 5.25E-05 | 1.88E-09 | 1.32E-05 |
| 9OH-10KOM | 2.33E-05 | 9.33E-05 | 1.78E-05 |
| 9,10-diHOM | 0.011383 | 2.64E-06 | 0.00038379 |
| 12,13-EpOM | 2.01E-06 | 0.00025134 | 0.0012045 |
| 9,10-EpOD | 0.0009166 | 0.0003695 | 0.0012272 |
| 13HOT | 0.00079993 | 0.011281 | 0.0017852 |
| 9KOT | 0.18499 | 1.33E-05 | 0.0018781 |
| 9OH-12KOD | 0.067634 | 0.0035867 | 0.0019872 |
| 9OH-12KOM | 0.00098776 | 0.66639 | 0.0029024 |
| 9HOT | 9.33E-05 | 0.00064857 | 0.0032057 |
| 12COOH-JA-Ile | 8.96E-05 | 2.79E-08 | 0.0042353 |
| 9,12,13-THOM | 2.33E-05 | 8.63E-07 | 0.0045029 |
| Azelaic acid | 0.0017164 | 0.0070627 | 0.0066642 |
| JA | 0.21857 | 3.61E-06 | 0.0098526 |
| 9,10-EpOM | 0.0004878 | 0.10359 | 0.013785 |
| 10-OPEA | 0.92868 | 1.66E-13 | 0.021106 |
| 9HOD | 2.33E-05 | 7.34E-05 | 0.03068 |
| 9,10,13-THOM | 0.00080916 | 0.00017003 | 0.034944 |
| 12,13-diHOM | 0.0070657 | 0.011281 | 0.058542 |
| 9KOD | 0.014311 | 0.32323 | 0.081768 |
| 9,10,11-THOD | 0.29141 | 0.003249 | 0.1146 |
| 9,12,13-THOD | 0.0070657 | 0.0021867 | 0.13832 |
| 13OH-12KOM | 0.0037863 | 0.63177 | 0.17681 |
| 2OH-palmitic acid | 0.31927 | 0.027588 | 0.18751 |
| coumaric acid | 0.18499 | 0.00014817 | 0.20024 |
| Traumatic acid | 0.00058756 | 0.49937 | 0.26403 |
| OPC4:0 | 0.088913 | 0.028862 | 0.27839 |
| 9,10,13-THOD | 0.0014892 | 0.033134 | 0.40395 |
| 12-OPDA | 0.036776 | 0.10359 | 0.4506 |
| 13HOD | 2.33E-05 | 0.95453 | 0.49474 |
| 10HOD | 2.01E-06 | 0.027588 | 0.56048 |
| OPC8:0 | 0.0041769 | 0.39852 | 0.73973 |

**Supplementary Table 3. Two-way ANOVA (genotype×time) statistical analysis of tested oxylipins.**
