# Supplementary material for: Transcriptome and Oxylipin Profiling Joint Analysis Reveals Opposite Roles of 9-Oxylipins and Jasmonic Acid in Maize Resistance to Gibberella Stalk Rot
Source: Front Plant Sci. 2021 Sep 7;12:699146. doi: 10.3389/fpls.2021.699146 (PMC8454893; doi:10.3389/fpls.2021.699146)
Supplement: Supplementary Table 4 — Content fold change of oxylipins identified in B73, lox5-3, and W438 at 24 and 48 hai. The fold changes of oxylipins were calculated based on the contents in infected samples over that in control samples at 24 and 48 hai, respectively. [file Table_4.DOCX]

**Supplementary Table 4. Content fold change of tested oxylipins at 24 and 48 hai in B73, *lox5-3* and W438.**

| **Oxylipins** | **B73_F24** | **B73_F48** | **lox5_F24** | **lox5_F48** | **W438_F24** | **W438_F48** |
| --- | --- | --- | --- | --- | --- | --- |
| 10HOD | 0.608517 | 0.724224 | 1.024859 | 1.205563 | 0.801379 | 0.586947 |
| 10-OPEA | 4.692319 | 21.83194 | 14.77204 | 95.01152 | 6.734333 | 10.03377 |
| 12,13-diHOM | 0.814192 | 1.555133 | 1.425424 | 3.611759 | 0.668818 | 1.639426 |
| 12,13-EpOD | 0.954094 | 0.635821 | 1.1827 | 3.020009 | 1.390618 | 1.492178 |
| 12,13-EpOM | 0.842664 | 0.952952 | 1.36699 | 1.98997 | 1.561577 | 1.834331 |
| 12OH-JA | 1.306931 | 1.018531 | 1.635438 | 1.22138 | 1.022476 | 1.468016 |
| 12OH-JA-Ile | 72.32885 | 161.6282 | 1.526718 | 8.591533 | 1.402516 | 2.210402 |
| 12-OPDA | 0.94071 | 1.115212 | 0.768851 | 1.29034 | 0.910515 | 1.358802 |
| 13HOD | 0.812217 | 0.594865 | 1.106637 | 1.261056 | 1.088902 | 1.049185 |
| 13HOT | 1.012898 | 0.651614 | 1.285052 | 1.508701 | 1.973136 | 1.891125 |
| 9KOD | 1.185822 | 0.675236 | 1.181879 | 1.515533 | 2.337488 | 1.684687 |
| 13OH-12KOD | 0.75555 | 0.671178 | 0.947686 | 0.60489 | 0.853523 | 1.227377 |
| 13OH-12KOM | 0.764928 | 0.677358 | 0.612618 | 0.983382 | 1.267038 | 1.396106 |
| 2HOD | 0.506659 | 1.425317 | 2.043737 | 1076.559 | 6.391359 | 1.168875 |
| 2OH-palmitic acid | 0.881427 | 1.702605 | 0.908006 | 3.037863 | 1.011142 | 1.179319 |
| 9,10,11-THOD | 0.895411 | 12.00971 | 1.223685 | 5.188075 | 1.680577 | 0.891272 |
| 9,10,13-THOD | 1.500935 | 0.661689 | 1.173073 | 1.658896 | 1.507163 | 1.251961 |
| 9,10,13-THOM | 0.715192 | 1.368361 | 0.972236 | 3.554408 | 0.955181 | 1.468619 |
| 9,10-diHOM | 0.530212 | 5.095057 | 1.889607 | 14.23768 | 0.580668 | 1.206889 |
| 9,10-EpOD | 0.985402 | 0.789177 | 1.705397 | 1.626127 | 1.794611 | 1.528115 |
| 9,10-EpOM | 0.851564 | 0.714147 | 1.653272 | 1.769697 | 1.674054 | 2.075664 |
| 9,12,13-THOD | 1.491731 | 0.553552 | 1.306019 | 1.063846 | 1.704426 | 1.228854 |
| 9,12,13-THOM | 0.890303 | 1.475667 | 1.11871 | 2.9987 | 1.243594 | 1.565953 |
| 9HOD | 0.57083 | 0.97718 | 1.704094 | 2.688266 | 0.804354 | 1.209748 |
| 13KOD | 1.03627 | 0.611203 | 0.9521 | 1.034313 | 0.728856 | 0.870202 |
| 9KOT | 1.140607 | 0.818083 | 1.394657 | 2.223348 | 2.227744 | 2.053639 |
| 9OH-10KOD | 0.668179 | 0.855673 | 2.33857 | 3.365238 | 1.04004 | 1.717762 |
| 9OH-10KOM | 0.49962 | 0.861958 | 3.919369 | 13.20482 | 2.030588 | 2.308006 |
| 9OH-12KOD | 0.645585 | 0.541844 | 1.215587 | 0.643786 | 1.391452 | 1.50201 |
| 9OH-12KOM | 0.587822 | 0.70202 | 0.945904 | 0.976651 | 0.781533 | 2.159907 |
| Azelaic acid | 0.851493 | 0.799309 | 1.297022 | 1.355353 | 1.066977 | 1.311981 |
| CA | 1.701523 | 146.5333 | 1.329394 | 189.8773 | 1.068558 | 2.529714 |
| coumaric acid | 1.200002 | 3.574714 | 0.853651 | 4.221729 | 1.709804 | 1.383522 |
| JA | 8.420401 | 12.20418 | 5.571059 | 29.85332 | 1.687709 | 5.926137 |
| JA-Ile | 22.73087 | 43.15212 | 14.76955 | 43.50453 | 0.906577 | 2.720443 |
| OPC4:0 | 2.050935 | 7.020197 | 1.010865 | 11.61089 | 1.412389 | 1.573051 |
| 12COOH-JA-Ile | 3.441657 | 17.58564 | 1.19395 | 7.427941 | 2.343037 | 2.630094 |
| 15,16-diHOD | 0.86363 | 1.012442 | 0.930545 | 0.865671 | 0.721291 | 1.254382 |
| 15,16-EpOD | 1.067704 | 0.602762 | 0.96833 | 1.096566 | 0.920396 | 0.902166 |
| p9OH-Tan | 0.710328 | 0.809644 | 0.978708 | 0.81818 | 1.261034 | 1.154668 |
| OPC8:0 | 1.058441 | 0.625376 | 0.678448 | 1.016199 | 0.975041 | 0.81582 |
| OTD | 1.065379 | 0.899377 | 1.310219 | 0.978436 | 1.547815 | 1.660674 |
| 9HOT | 1.557882 | 0.765999 | 1.622975 | 1.069135 | 4.175438 | 2.039831 |
| Traumatic acid | 0.780267 | 1.088977 | 1.396888 | 1.535408 | 0.961923 | 1.129278 |
